# Dehydroepiandrosterone Heightens Aggression and Increases Androgen Receptor and Aromatase mRNA Expression in the Brain of a Male Songbird

**DOI:** 10.1111/jne.12443

**Published:** 2016-12-09

**Authors:** D. W. Wacker, S. Khalaj, L. J. Jones, T. L. Champion, J. E. Davis, S. L. Meddle, J. C. Wingfield

**Affiliations:** ^1^ School of STEM (Division of Biological Sciences)University of Washington BothellBothellWAUSA; ^2^ BiologySeattle UniversitySeattleWAUSA; ^3^ Biology DepartmentRadford UniversityRadfordVAUSA; ^4^ The Roslin Institute and Royal (Dick) School of Veterinary StudiesUniversity of EdinburghEdinburghUK; ^5^ College of Biological SciencesUniversity of California DavisDavisCAUSA

**Keywords:** dehydroepiandrosterone, aromatase, androgen receptor, aggression, sparrow

## Abstract

Dehydroepiandrosterone (DHEA) is a testosterone/oestrogen precursor and known modulator of vertebrate aggression. Male song sparrows (*Melospiza melodia morphna*) show high aggression during breeding and nonbreeding life‐history stages when circulating DHEA levels are high, and low aggression during molt when DHEA levels are low. We previously showed that androgen receptor and aromatase mRNA expression are higher during breeding and/or nonbreeding in brain regions associated with reproductive and aggressive behaviour, although the potential role of DHEA in mediating these seasonal changes remained unclear. In the present study, nonbreeding male song sparrows were captured and held in the laboratory under short days (8 : 16 h light/dark cycle) and implanted with s.c. DHEA‐filled or empty (control) implants for 14 days. DHEA implants increased aggression in a laboratory‐based simulated territorial intrusion. Brains of DHEA‐implanted birds showed higher aromatase mRNA expression in the preoptic area (POA) and higher androgen receptor mRNA expression in the periventricular nucleus of the medial striatum (pvMSt) and ventromedial nucleus of the hypothalamus. The DHEA‐induced increases in aromatase expression in the POA and androgen receptor expression in the pvMSt are consistent with previously reported seasonal increases in these markers associated with naturally elevated DHEA levels. This suggests that DHEA facilitates seasonal increases in aggression in nonbreeding male song sparrows by up‐regulating steroid signalling/synthesis machinery in a brain region‐specific fashion.

Dehydroepiandrosterone (DHEA) is an androgen produced in the cortical cells of the adrenal gland, the testes, as well as *de novo* in the brain 1, 2, 3. It is a known modulator of neurophysiology and behaviour, including aggression 2, 4, 5. Regardless of whether it is of adrenal or neural origin, once in the brain, DHEA can be readily transformed into more bioactive androgens (e.g. testosterone and 5α‐dihydrotestosterone) and oestrogens (e.g. 17β‐oestradiol). These steroids may then regulate changes in behaviour 6, 7, 8, 9. DHEA and/or its sulphate ester, DHEA‐S may also exert direct effects on behaviour because they bind a variety of receptors, including oestrogen receptor β, the androgen receptor, the sigma‐1 receptor and the GABA_A_ receptor 10, 11, 12, 13. Whether DHEA can directly modulate nuclear sex steroid receptors at physiologically and behaviourally relevant concentrations is still debatable 10, 13, 14, 15.

DHEA is produced in a variety of vertebrates, including birds 3, mammals 16 and humans 17. It is also utilised as an over‐the‐counter supplement and multiple health and therapeutic benefits have been reported 18, 19. DHEA is classified as a performance enhancing drug and is listed as a banned substance by the World Anti‐Doping Agency, partly as a result of concerns about the potential role of exogenous androgens in facilitating aggression 20. Exogenous DHEA has been shown to increase aggression in animal studies 4, 5, although its potential role as a modulator of human aggression is less clear. Circulating DHEA levels are higher in imprisoned criminals than healthy nonconvicts, although whether this difference has more to do with criminality or imprisonment is debatable 21. Elevated levels of DHEA‐S, the dominant form of DHEA in human plasma, are associated with childhood conduct disorder, a condition linked with adult antisocial behaviour 22, 23. DHEA‐S levels are positively correlated with the ‘intensity of aggression and delinquency’ 23 and ‘disturbance‐to‐others’ 22 in these human studies.

The Pacific Northwest Song Sparrow (*Melospiza melodia morphna*) is an excellent model for better understanding the mechanisms underlying DHEA‐induced vertebrate aggression. Male song sparrows are territorial and show varying, yet predictable levels of aggression throughout the year 24. Seasonal increases in male song sparrow aggression are associated with concomitant increases in circulating DHEA 3 and administration of DHEA increases song sparrow aggression in both laboratory and field settings 4, 5. DHEA likely acts via both acute and longer‐term mechanisms to alter aggressive territoriality in the male song sparrow. Previous work has demonstrated that DHEA can be rapidly converted to bioactive androgens in response to an aggressive conspecific challenge 7 and seasonal changes in DHEA profiles are associated with alterations in the brain‐region specific expression of androgen receptor and aromatase mRNA 25. The effects of exogenous DHEA and its metabolites on neural steroid signalling and synthesis machinery, as well as how these changes may relate to aggression, have yet to be examined thoroughly.

In the present study, we assessed changes in aggression and neural androgen receptor, as well as aromatase mRNA expression, in the brains of nonbreeding male song sparrows given a chronic (14 days) administration of DHEA. Previous studies suggest that heightened endogenous DHEA and testosterone levels are associated with increases in neural androgen receptor and/or aromatase mRNA expression in specific brain regions in Emberizid sparrows 25, 26. Specifically, nonbreeding and/or breeding male song sparrows (high DHEA) had higher androgen receptor mRNA levels than molting males (low DHEA) in the pre‐optic area (POA) and periventricular nucleus of the medial striatum (pvMSt) and higher aromatase expression in the POA, medial preoptic area/medial division of the bed nucleus of the stria terminalis (mPOA/BSTM) and ventromedial nucleus of the hypothalamus (VMH) 25. Fraley *et al*. 26 reported that administration of testosterone to captive Gambel's white‐crowned sparrows (*Zonotrichia leucophrys gambelii*), held under long days indicative of their breeding season, led to increases in androgen receptor mRNA expression in the lateral division of the bed nucleus of the stria terminalis (BSTL). Therefore, we predicted that DHEA administration would increase androgen receptor and/or aromatase mRNA expression in these brain regions, and also that these changes would be concomitant with increases in aggressive behaviour.

## Materials and methods

### Animals and housing

All procedures were performed in accordance with University of Washington Animal Care and Use Committee guidelines and the National Institutes of Health Guide for the Care and Use of Laboratory Animals. Nonbreeding male song sparrows were captured in the autumn at multiple sites in Western Washington State. Each bird was lured into a mist net with playback of conspecific song within its territory. Birds were group housed in outdoor aviaries, then placed in individual cages (35 × 40 × 42 cm) and held under short days (8: 16 h light/dark cycle; 8L : 16D) at 20 °C for the duration of the experiment. Each bird was separated from its closest neighbors by opaque partitions.

### DHEA administration

Administration of DHEA was conducted as described by Wacker *et al*. 5. Briefly, male song sparrows were anaesthetised with a mixture of isoflurane and oxygen gases. An empty (n = 6) or DHEA‐filled (n = 6) (D4000; Sigma, St Louis, MO, USA) silastic implant (Baxter Healthcare, Deerfield, IL, USA; effective length 7 mm; diameter 1.65 mm inner/0.76 mm outer), sealed on both ends with silicone, was implanted s.c. on the left flank of each animal. Previous work has demonstrated that this procedure elevates circulating DHEA to high but physiologically relevant levels, which leads to increases in aggressive behaviour in nonbreeding male song sparrows 4, 5.

### Behavioural testing and tissue collection

Aggression was assessed after 14 days of implantation utilising a laboratory‐based simulated territorial intrusion (STI) 5, 27. Briefly, each male in its home cage was moved into a behavioural testing chamber and placed next to a caged, novel, nonbreeding male song sparrow; cages were initially separated by a curtain. Each focal bird was allowed to equilibrate for 10 min. At the start of the laboratory‐based STI, the curtain was raised, exposing the live decoy to the focal bird. At the same time, playback of recorded conspecific male song was initiated through a speaker located on the far side of the decoy bird's cage. Audio and video of the focal bird's reaction was recorded for analysis. Video was taken through a one‐way glass window, where the observer was not observable by either the focal or decoy bird. Video was analysed on a second‐by‐second basis by an observer who was blind to treatment, and aggressive behaviours and the latency to the first aggressive response were recorded for each bird. A total aggression score was calculated by scoring aggressive postures, movements and vocalisations, and then summing these scores over the 10‐min testing period 5, 28. At the end of each laboratory‐based STI, the focal bird was immediately deeply anaesthetised with an i.m. injection of pentobarbital (Nembutal Sodium Solution; Abbot Laboratories, Abbot Park, IL, USA; 50 mg/ml) and decapitated. Blood was collected for subsequent radioimmunoassay and the brain was removed, flash‐frozen on dry ice and stored at −80 °C.

### *In situ* hybridisation

*In situ* hybridisation protocols were exactly as described by Wacker *et al*. 25. Briefly, brains were sectioned coronally on a cryostat to 15 μm and thaw‐mounted onto RNase‐free poly‐l‐lysine‐coated glass slides. A synthetic oligoprobe (cDNA) for aromatase was created (GeneDetect, Bradenton, FL, USA) from a published zebra finch (*Taeniopygia guttata*) aromatase 3188‐bp sequence. The probe hybridises to nucleotides 688–735 located within the coding sequence of ZA1 (GenBank L81143.1) 29. This oligoprobe was previously used to successfully label aromatase mRNA in song sparrows 25. The probe was 3′‐labelled with ^35^S‐dATP using terminal deoxynucleotidyl transferase and then purified with spin columns (QIAquick nucleotide removal kit; Qiagen, Crawley, West Sussex, UK). Sections were hybridised with radiolabelled oligoprobe overnight at 37 °C in humidified chambers, rinsed and allowed to air dry.

A 363‐bp fragment encoding the parts of the hinge and ligand‐binding domains of the white‐crowned sparrow (*Zonotrichia leucophrys*) androgen receptor 30 was used to construct a cDNA, which was subcloned into pBSK. This riboprobe was previously used to successfully label androgen receptor mRNA in song sparrows 25. The ^35^S‐labelled antisense or sense riboprobe was then applied at a concentration of 106 cpm/slide in standard riboprobe hybridisation buffer and incubated for 16–18 h at 55 °C in humidified chambers. Slides were incubated in ribonuclease A rinsed, and dehydrated in a graded ethanol series containing ammonium acetate.

Following hybridisation, all slides were dipped in liquid autoradiographic emulsion (Kodak type NTB‐3; Anachem, Bedfordshire, UK), dried, and stored with desiccant in light‐proof boxes at 4 °C for 4 weeks for aromatase mRNA and 6 weeks for androgen receptor mRNA. Slides were developed (D19; Eastman Kodak, Rochester, NY, USA), counterstained with haematoxylin and eosin (H&E) and mounted using DPX (Merck‐BDH; Lutterworth, Leicestershire, UK). There was no detectable hybridisation signal with any of the sense probes or after RNase A (30 mg/ml) pretreatment.

### Quantification of relative silver grain density

**Figure 1:**
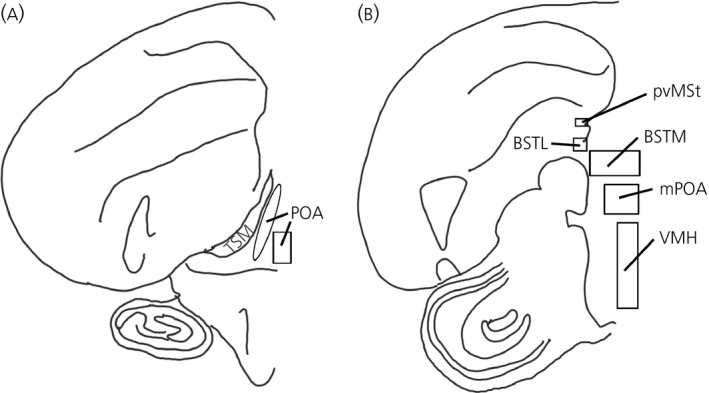
Diagrams of brain regions sampled, based on the canary atlas of Stokes *et al*. 31 with modifications based on the differences in the song sparrow brain as outlined in Wacker *et al*. 25. Rostral–caudal approximations are as follows (though brain regions extend into adjacent sections): (a) ~A2.6 includes the preoptic area (POA). Aromatase mRNA expression was observed in two distinct regions: one running along the septopalliomesencephalic tract (TSM) and another along the midline. Androgen receptor mRNA expression was more ubiquitous across the whole area. (b) ~A1.2 includes the lateral part of the bed nucleus of the stria terminalis (BSTL), medial part of the bed nucleus of the stria terminalis (BSTM), medial preoptic area (mPOA), ventromedial nucleus of the hypothalamus (VMH) and periventricular nucleus of the medial striatum (pvMSt).

**Figure 2:**
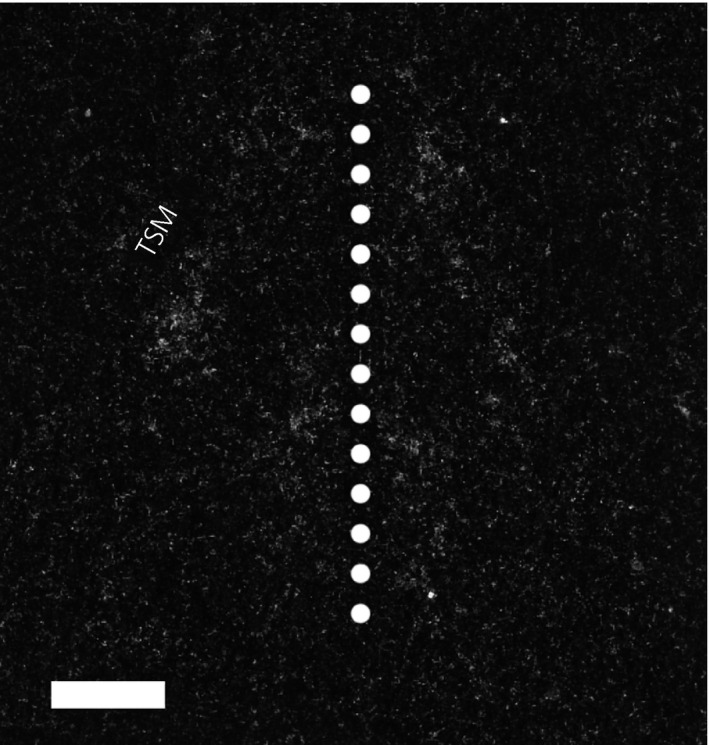
Aromatase mRNA expression in the preoptic area. Expression was observed in two distinct regions: one running medial to the septopalliomesencephalic tract (TSM) and another, more ventrally, along the midline (dotted line). Scale bar = 250 μm.

**Figure 3:**
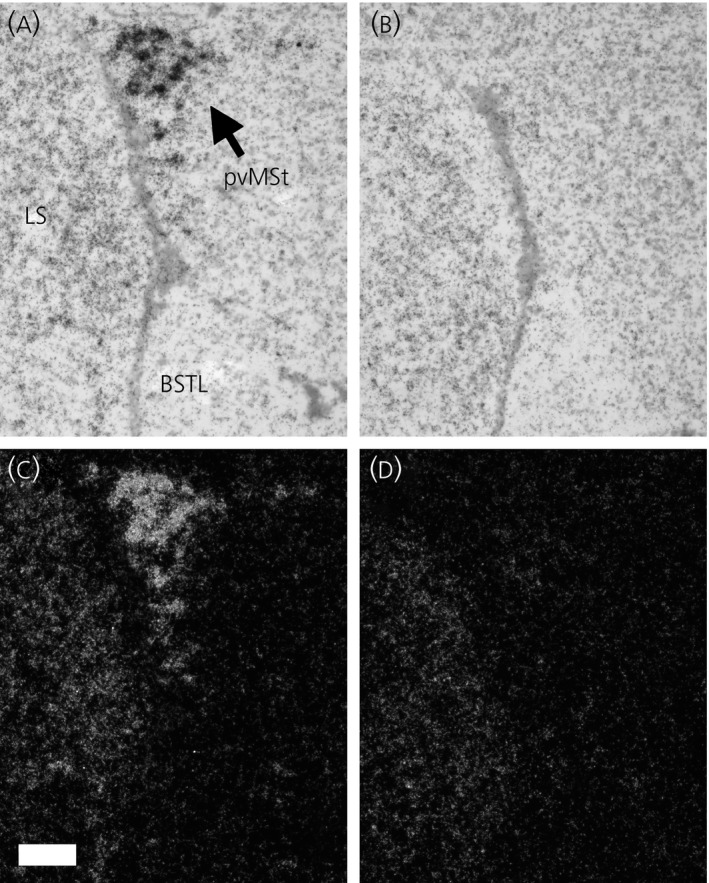
Androgen receptor mRNA expression in the periventricular nucleus of the medial striatum (pvMSt) of nonbreeding male song sparrows with dehydroepiandrosterone (DHEA) (a, bright field; c, dark field) or control/empty implants (b, bright field; d, dark field). Note the relative lack of androgen receptor mRNA in what is considered the lateral part of the avian bed nucleus of the stria terminalis (BSTL) in both DHEA‐implanted and control birds. LS, lateral septum. Scale bar = 100 μm.

Brain regions were defined employing a canary brain atlas 31 corrected for current avian brain nomenclature 32, as well as published aromatase and androgen receptor mRNA distributions in multiple avian species 8, 25, 26, 33, 34, 35 (Fig. 1). H&E co‐staining revealed neuroanatomical landmarks, allowing for the consistent and accurate delineation of boundaries for each brain region examined. Delineation of each brain region was performed as described previously by Wacker *et al*. 25 with certain differences. We observed two mostly separate bilateral bands of aromatase mRNA expression in the POA, with one band running along the midline and another ventromedial to the septopalliomesencephalic tract (TSM; formerly septomesencephalic tract, TrSM) (Fig. 2). These subregions of POA were therefore assessed separately for aromatase mRNA in the present study. The mPOA includes regions ventral to the anterior commissure. After the anterior commissure disappears caudally, the mPOA and BSTM combine to form a continuous band of aromatase and androgen receptor mRNA expressing cells. In the present study, we refer to these areas collectively as mPOA. Areas of hybridisation dorsal to the anterior commissure, ventral to the lateral septum and ventromedial to the BSTL are defined as BSTM. The BSTL lies along the lateroventral edge of the ventricle opposite of the lateral septum, whereas the pvMSt 26 is both distinct and separate and lies dorsal to what is traditionally identified as BSTL (Fig. 3).

Hybridisation densities were quantified under dark field as described previously 25. Briefly, images were taken using a DS‐Qi1Mc (Nikon Instruments Inc., Melville, NY, USA) digital camera attached to an Eclipse 80‐i microscope (Nikon Instruments Inc.) at × 40 or × 100 magnification. Photomicrographs were captured and the silver grain density was quantified by measuring the mean pixel density in a sampled area in each brain region using nis‐elements br (Nikon Instruments Inc.). Sampling areas for each nucleus were chosen such that at least 95% of the region of interest was included in each measurement, and these sampling areas were kept consistent for each brain region across all individuals. Each measurement was divided by the background pixel density of a control area that lacked positive hybridisation within the same section. A value of 1 was subtracted from this value to calculate the proportion of pixel density in the region of interest over background pixel density, such that, when the signal equaled background, a value of 0% would be reported. This number was multiplied by 100 to yield relative silver grain density (percentage over background).

### Hormone assays

Hormone assays were conducted as described by Soma and Wingfield 2001 3 and Wacker *et al*. 5. Briefly, steroids were extracted from plasma samples with dichloromethane, and DHEA and testosterone were separated under nitrogen pressure on diatomaceous earth/glycol columns and measured via radioimmunoassay, using [^3^H]‐DHEA (catalogue number NET814250UC; Perkin Elmer, Waltham, MA, USA) or [^3^H]‐testosterone (catalogue number NET553250UC; Perkin Elmer), respectively 3, 5. All samples were run in duplicate, using polyclonal rabbit anti‐DHEA (catalogue number 20‐DR86; Fitzgerald, Acton, MA, USA) or polyclonal rabbit anti‐testosterone (catalogue number 20R‐TR018W; Fitzgerald) antisera, respectively. The lower detection limits were 0.15 ng/ml for DHEA and 0.29 ng/ml for testosterone. One control value for DHEA and one control value for testosterone were lower than their respective detection limits, and so were assigned the detection limit as their value for calculation of the mean ± SE, as well as for subsequent statistical comparisons. Intra‐assay variations were 16% for DHEA and 9% for testosterone.

### Statistical analysis

Because DHEA implants have previously been shown to increase both circulating DHEA and testosterone levels, one‐tailed t‐tests were used to examine the effects of the DHEA implants on these hormone levels. Because DHEA implants have been previously shown to increase nonbreeding song sparrow aggression in laboratory‐based STIs, one‐tailed t‐tests were used to examine the effects of DHEA on both the total aggression score and latency to aggression 5.

In brain regions where seasonally elevated DHEA levels are associated with higher androgen receptor and/or aromatase mRNA expression 25, we utilised one‐tailed t tests to assess our predictions that exogenous DHEA would increase aromatase mRNA expression in the POA, VMH and mPOA, as well as increase androgen receptor mRNA expression in the POA and pvMSt. Two‐tailed tests were used to assess DHEA effects on aromatase mRNA expression in the BSTL and BSTM, as well as androgen receptor expression in the BSTM and VMH, because these brain areas were either not previously examined (BSTL, BSTM) or seasonal changes were not previously detected (androgen receptor expression in VMH).

When values were not normally distributed (Shapiro–Wilk W test) and/or when variances across groups were heterogeneous (O'Brien test), natural log transformations were used to achieve normality and homogeneous variances across groups 36. All values are reported as the mean ± SE.

## Results

### DHEA implants increase circulating DHEA and testosterone levels

DHEA, implanted for 14 days, significantly increased both circulating DHEA [ln(DHEA) t_0.05(1),10_ = 8.96; P* *≤ 0.0001] and testosterone levels [t_0.05(1),10_ = 5.75; P* *≤ 0.0001] in nonbreeding male song sparrows. DHEA levels for DHEA‐implanted birds were 2.17 ± 0.45 ng/ml compared to 0.22 ± 0.03 for control birds. Testosterone levels for DHEA‐implanted birds were 2.06 ± 0.26 ng/ml compared to 0.48 ± 0.09 ng/ml for control birds.

### Exogenous DHEA increases aggression

**Figure 4:**
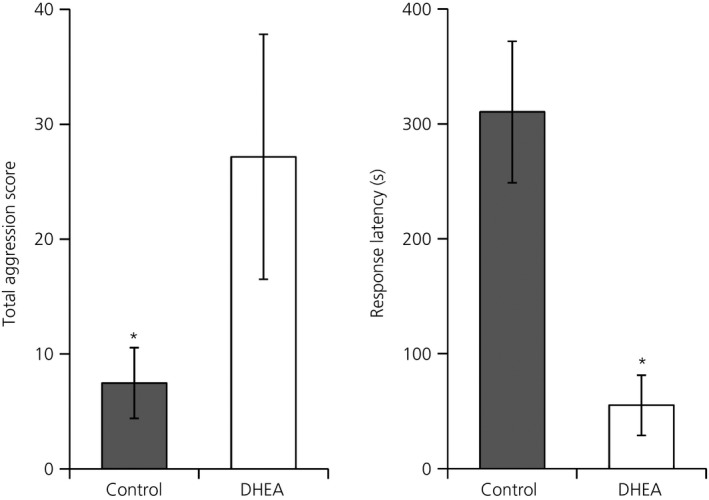
Dehydroepiandrosterone (DHEA) administration increases aggression in male song sparrows. Nonbreeding male songs sparrows, held under short days (8L : 16D) implanted with DHEA‐filled silastic implants for 14 days showed higher total aggression scores and a shorter latency to show an aggressive behaviour than birds with empty implants. DHEA, n = 6; Control, n = 6. Asterisks denote significant differences at P ≤ 0.05.

DHEA significantly increased total aggression scores in nonbreeding male song sparrows [ln(total aggression score) t_0.05(1),10_ = 2.50; P* *≤* *0.05] (Fig. 4). Average total aggression scores for DHEA‐implanted birds were 27 ± 11 compared to 7 ± 3 for control birds. DHEA administration also decreased the latency to the first instance of an aggressive behaviour in these tests [ln(response latency) t_0.05(1),10_ = −3.95; P* *≤* *0.005] (Fig. 4). Average latencies to aggression for DHEA‐implanted birds were 55 ± 25 s compared to 310 ± 62 s for control birds.

### Exogenous DHEA alters aromatase mRNA expression in a brain‐region specific fashion

**Figure 5:**
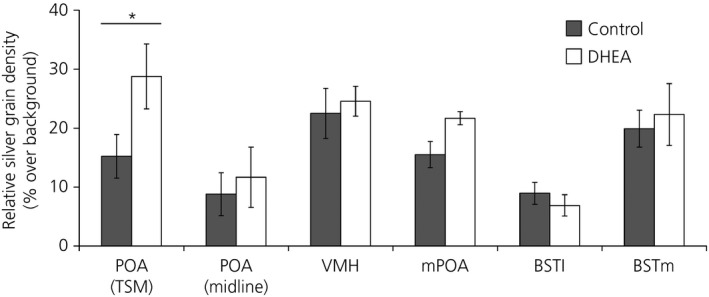
Dehydroepiandrosterone (DHEA) led to significantly higher aromatase mRNA expression along the septopalliomesencephalic tract (TSM), but not along the midline, in the preoptic area (POA). DHEA did not alter aromatase mRNA expression in any other brain region examined. POA (TSM), ventromedial nucleus of the hypothalamus (VMH), medial preoptic area (mPOA), lateral part of the bed nucleus of the stria terminalis (BSTL), medial part of the bed nucleus of the stria terminalis (BSTM): DHEA, n = 6; control, n = 6; POA (midline): DHEA, n = 4; Control, n = 5. Graphs depict the mean ± SEM. Asterisks denote significant differences at P ≤ 0.05.

DHEA significantly increased aromatase mRNA expression in the subdivision of POA ventromedial to the TSM [t_0.05(1),10_ = 2.04; P* *≤* *0.05] but not in the subdivision of the POA along the midline [t_0.05(1),7_ = 0.44; P* *=* *0.34] (Fig. 5). DHEA administration did not alter aromatase mRNA expression in the BSTL [t_0.05(2),10_ = −0.80; P* *=* *0.44], BSTM [t_0.05(2),10_ = 0.39; P* *=* *0.70], mPOA [t_0.05(1),10_ = 1.15; P* *=* *0.14] or VMH [t_0.05(1),10_ = 0.42; P* *=* *0.34].

### Exogenous DHEA alters androgen receptor mRNA expression in a brain‐region specific fashion

**Figure 6:**
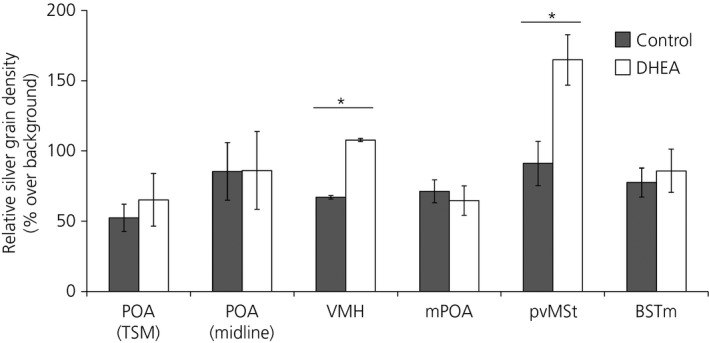
Dehydroepiandrosterone (DHEA) led to significantly higher androgen receptor mRNA expression in ventromedial nucleus of the hypothalamus (VMH) and periventricular nucleus of the medial striatum (pvMSt), but not in other brain regions examined. Graphs depict the mean ± SEM. Asterisks denote significant differences at P ≤ 0.05. Preoptic area along the septopalliomesencephalic tract (POA TSM), POA (midline): DHEA, n = 5; control, n = 5; VMH, medial pre‐optic area (mPOA), pvMSt, medial part of the bed nucleus of the stria terminalis (BSTM): DHEA, n = 6; control, n = 6. Graphs depict the mean ± SEM. Asterisks denote significant differences at P ≤ 0.05.

DHEA significantly increased androgen receptor mRNA expression in the pvMSt [t_0.05(1),10_ = 3.08; P* *≤ 0.01] and VMH [t_0.05(2),10_ = 2.52; P* *≤ 0.05] (Fig. 6). Apart from expression in the nearby pvMSt, there was little to no androgen receptor mRNA in the BSTL in DHEA or control birds, so this region was not analysed. DHEA administration did not alter androgen receptor expression in the subdivision of the POA just ventral to the TSM [ln(POA_TSM_) t_0.05(1),8_ = 0.72; P* *=* *0.25], or the subdivision of the POA along the midline [t_0.05(1),8_ = 0.03; P* *=* *0.49], BSTM [t_0.05(2),10_ = 0.45; P = 0.66] or mPOA [t_0.05(2),10_ = −0.50; P = 0.63].

## Discussion

DHEA is an important modulator of social behaviours, including aggression, in many vertebrates, including in humans 3, 5, 22, 23. Interestingly, circulating DHEA/DHEAS levels are relatively low in laboratory mice and rats 37. Male song sparrows provide an ideal alternative model for the study of DHEA‐induced effects on vertebrate brain and behaviour. Male song sparrows have higher circulating levels of DHEA during both their breeding and nonbreeding life‐history stages when aggression is high compared to their molt when aggression is low 3, 24. Seasonally changing androgen levels in male song sparrows are concomitant with alterations in androgen receptor and aromatase mRNA expression in brain regions that regulate vertebrate sexual behaviour and aggression 25, 38, 39, and administration of DHEA facilitates aggressive behaviours such as territorial song and threatening postures in this species 4, 5. In this study, we demonstrate that administration of exogenous DHEA increases aggression in nonbreeding male song sparrows, while simultaneously increasing androgen receptor and aromatase mRNA expression in brain regions of the social decision‐making network, a set of interconnected brain regions that modulate a myriad of critical vertebrate social behaviours 38, 39. This suggests that increases in aggression associated with exogenous DHEA are mediated, at least in part, by alteration of steroid synthesis and signalling pathways in this neural network.

Previous work has demonstrated that 3β‐hydroxysteroid dehydrogenase (3β‐HSD), the enzyme that converts DHEA to androstenedione, can be rapidly activated in the brains of nonbreeding male song sparrows stimulated by an aggressive encounter 7. This activation can ultimately lead to the production of more bioactive sex steroids, such as testosterone and 17β‐oestradiol, which can bind to androgen and oestrogen receptors, respectively, allowing a bird to quickly respond to social challenge. This binding may be to nuclear sex steroid receptors, such as the androgen receptor or oestrogen receptor α or β, and/or to nongenomic oestrogen receptors, which may affect nonbreeding male aggression in song sparrows 40. Such activational effects, however, do not preclude potential longer‐term effects of DHEA on seasonal changes in aggression.

Because circulating DHEA levels, 3β‐HSD and aromatase activity, and aromatase and androgen receptor mRNA expression all show longer‐term seasonal change in male song sparrows, the potential for seasonally‐organised steroidal effects on behaviour cannot be overlooked 3, 8, 25. Although the phrase ‘organisational effects of steroids’ is often used to describe permanent developmental alterations of anatomy and physiology, life‐history changes in neural steroid signalling machinery, such as those described in Wacker *et al*. 25, may remain relatively static across a particular season, and so do not fit a strict definition of activational effects. DHEA can rapidly activate these DHEA‐organised circuits during specific social interactions, such as territorial disputes, thus having both activational and seasonally‐organised effects. This is consistent with the observation that heightened DHEAS levels are associated with an increased proclivity towards aggression in some humans 22, 23. In the present study, we show that exogenous DHEA can re‐organise components of neural networks that modulate social behaviour in male song sparrows. This opens up the possibility that exogenous DHEA and/or DHEAS may induce longer‐term changes to neural signalling pathways affecting social behaviours in humans. This is of particular clinical interest because DHEA is readily available as an over‐the‐counter supplement and prescribed to both men and women for a variety of reasons 15, 18, 19.

In the present study, chronic administration of DHEA to nonbreeding song sparrows elevated androgen receptor and aromatase mRNA expression in some of the same brain regions where seasonal differences were previously detected 25. DHEA levels are highest during breeding and nonbreeding versus molt in male song sparrows 3 and androgen receptor mRNA expression is highest during breeding in the POA and pvMSt 25. In the present study, administration of DHEA increased androgen receptor expression in the pvMSt but not in the POA. Interestingly, exogenous DHEA also increased androgen receptor expression in the VMH, where a seasonal difference was not detected. Aromatase mRNA expression is highest during breeding in the POA and mPOA/BSTM, and equally high in the VMH during breeding and nonbreeding (versus molt where it is lower) 25. In the present study, DHEA induced increased aromatase expression in the POA but not in mPOA, BSTM or VMH. The finding that there were some differences between the results of the present study and our previous seasonal comparison is not unexpected. In the present study, we only altered one variable, DHEA, whereas the seasonal movement through a series of life‐history stages involves changes to a myriad of morphological, physiological and behavioural attributes 41. Also, DHEA levels were not measured in our seasonal comparison 25, and so it is not possible to compare levels of this hormone across these studies. We previously demonstrated that very small changes in circulating androgen levels correlate with an up‐regulation of both aromatase and androgen receptor expression in a variety of brain regions 25. Thus, it is possible that the effects observed in the present study are at least partially mediated by increased testosterone derived from the metabolic conversion of administered DHEA. This is consistent with the prediction that DHEA would elicit at least some of its effects indirectly after conversion to more bioactive androgens or oestrogens.

The consistencies in androgen receptor and aromatase mRNA expression associated with naturally elevated DHEA levels and those induced by exogenous DHEA are perhaps more interesting than the differences. Increased androgen receptor expression in pvMSt and aromatase expression in POA are associated with both seasonally elevated endogenous and experimentally elevated exogenous DHEA levels 25. That the DHEA‐induced changes in pvMSt and POA were concomitant with increases in aggressive behaviour suggests that these brain regions are involved in the sex steroid‐dependent modulation of aggression in this species. The lack of a difference in aromatase mRNA expression in VMH in the present study is not unexpected because breeding and nonbreeding levels of this marker are equivalent and DHEA levels are equally high in both of these life‐history stages 3, 25. The finding of increased androgen receptor expression in VMH was unexpected but, as with the seasonal findings, it points to this brain region as a target for future examination.

The paucity of androgen receptor mRNA expression in BSTL was curious because most subdivisions of the rat BST are rich in androgen receptor message 42. We observed a high level of hybridisation in a region dorsal to that commonly considered the BSTL 31, 32, in an area we previously termed the pvMSt 25 (Fig. 3). Androgen receptor mRNA has been detected in this area in both zebra finches 35 and juncos (*Junco hyemalis*) 43. Although Satre *et al*. 43 also noted the location of this hybridisation as being dorsal to what is commonly considered the BST, Fraley *et al*. 26 identified this area as BSTL in white‐crowned sparrows. Many avian studies only divide the BST into lateral (BSTL) and medial (BSTM) parts 32, although other subdivisions of BST have been suggested more recently for nonsongbirds 44. The rat BST has been divided into fifteen subdivisions, all positive for androgen receptor mRNA expression 42. The pvMSt is possibly homologous to a subdivision of the BST that has yet to be properly characterised in birds, although subsequent studies are needed to validate this hypothesis. Such a finding would not be unexpected because BST is involved in the regulation of a variety of vertebrate social behaviours, including reproductive behaviour and aggression 45, 46, and androgen regulation of such behaviour would be predicted.

Another intriguing possibility is that cells expressing androgen receptor mRNA in the pvMSt represent newly born neurones bound for incorporation into the hippocampus or song control nuclei. The pvMSt is located within the larger lateral ventricular zone where neurogenesis is initiated 47, 48, 49 and both the hippocampus and HVC are rich in androgen receptors and comprise sites of seasonal incorporation of new neurones 49, 50. Although androgen receptor mRNA expression is not detected in HVC until 9–11 days after hatch in the zebra finch, during both embryonic development and on posthatch day 1, androgen receptor mRNA is already expressed in the lateral ventricular zone 35, 51. Although it remains unclear whether new neurones express androgen receptor in the proliferative zone of adult birds, these findings obtained in developing animals suggest that such expression is possible. Furthermore, it was recently shown that DHEA increases the number of bromodeoxyuridine positive (i.e. proliferating) cells in the song sparrow brain 49, which, along with our data, suggests that DHEA might facilitate the seasonal proliferation of androgen sensitive cells. Although circulating DHEA levels are no different during breeding and nonbreeding 3, brain‐region specific differences in DHEA utilisation may facilitate such changes.

In the present study, we show that chronic administration of DHEA increases circulating DHEA and testosterone levels, androgen receptor and aromatase mRNA expression in a brain region‐specific fashion, and aggression in nonbreeding male song sparrows. To our knowledge, the present study is the first to link exogenous DHEA administration with both changes in neural architecture and concomitant increases in vertebrate aggression.
